# DNA Double Strand Break Repair in Mitosis Is Suppressed by Phosphorylation of XRCC4

**DOI:** 10.1371/journal.pgen.1004598

**Published:** 2014-08-28

**Authors:** Susan P. Lees-Miller

**Affiliations:** Department of Biochemistry and Molecular Biology, University of Calgary, Calgary, Alberta, Canada; Duke University, United States of America

Cells are continuously subjected to DNA damage, the most cytotoxic form of which is the DNA double-strand break (DSB). DSBs arise from endogenous sources, such as collapsed replication forks, or can be caused by exogenous agents that include ionizing radiation, reactive oxygen species, and chemotherapeutic drugs such as topoisomerase II poisons [1]. During interphase, DSBs are repaired by one of two main pathways: classical non-homologous end joining (C-NHEJ) or homologous recombination repair (HRR) [2]. The fate of DSBs that arise during mitosis, however, has been poorly characterized. In this issue of *PLOS Genetics*, Shinohara and colleagues demonstrate that DSB induction in mitosis leads to the formation of anaphase bridges, a hallmark of genomic instability, and demonstrate that their formation requires primarily the C-NHEJ pathway [3].

C-NHEJ, which is active throughout interphase, is considered a rapid but error-prone pathway that rejoins DSB ends with minimal end processing. The main steps in C-NHEJ are detection of the DSB by the Ku heterodimer, followed by recruitment of end-processing factors and the DNA ligase IV-XRCC4-XLF complex. Regulation of C-NHEJ end processing is controlled by the DNA-dependent protein kinase catalytic subunit (DNA-PKcs) [4], [5]. HRR, on the other hand, occurs only in S and G2 and is initiated by the binding of the MRN complex (composed of Mre11, Rad50, and Nbs1), which is followed by MRN- and CtIP-mediated resection to create long 3′ overhanging ends required for Rad51-dependent strand invasion. Unlike C-NHEJ, HRR requires an undamaged DNA template for repair, usually the sister chromatid, and results in slower, but more precise, repair [6]. In addition to the two main repair pathways, DSBs can also be repaired by an error-prone, alternative end-joining pathway (Alt-NHEJ). Alt-NHEJ requires end-resection and involves CtIP, XRCC1, and DNA ligase III, but not the C-NHEJ factors [7].

DSBs initiate an elaborate signaling cascade that involves protein phosphorylation and ubiquitylation. This results in accumulation of proteins on chromatin surrounding the DSB to form DNA damage-induced foci and is required for regulation of repair and for cell cycle checkpoint arrest. Checkpoint signaling is initiated primarily by ataxia telangiectasia mutated (ATM)-dependent phosphorylation of histone H2AX, which in turn leads to recruitment of the checkpoint mediator MDC1 and the E3 ubiquitin ligases RNF8 and RNF168, as well as 53BP1 and BRCA1 [2], [8].

While repair of DSBs and cell cycle checkpoint arrest in interphase cells is relatively well understood, less is known regarding how cells respond to DSBs that are incurred when cells are in mitosis. This is of particular importance as errors in mitosis can lead to chromosome aberrations, genomic instability, polyploidy, or mitotic catastrophe [9], [10]. Early studies indicated that DSB repair pathways are suppressed in mitosis [11], and that DSBs originating in mitosis are not repaired until the subsequent G1 phase (reviewed in [12], [13]). In 2010, Jackson and colleagues reported that DSBs formed in mitosis initiate a partial DNA damage response in which ATM is activated, H2AX is phosphorylated, and MDC1 is recruited to foci [14]. Downstream recruitment of RNF8, RNF168, 53BP1, and BRCA1 fails to occur, however [14], [15]. Because 53BP1 promotes NHEJ [2], one mechanism for suppression of NHEJ in mitosis may be loss of 53BP1 from foci. Indeed, Durocher and colleagues recently showed that phosphorylation of RNF8 and 53BP1 by cyclin dependent kinase (CDK1) and the mitotic polo-like kinase 1 (PLK1) prevents their recruitment to DNA damage foci, thereby inactivating repair and minimizing opportunities to produce deleterious telomeric fusions [16].

**Figure 1:**
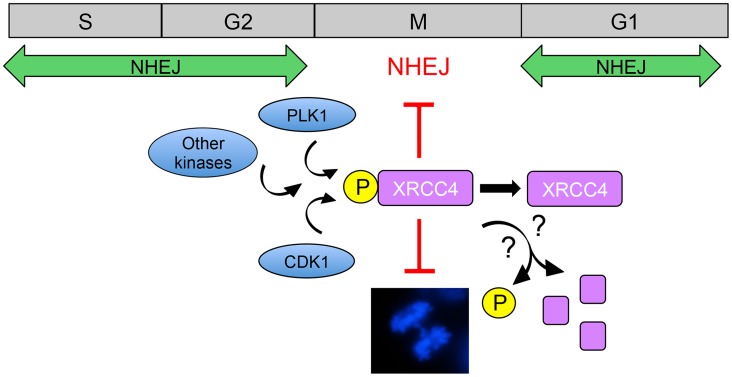
Model for suppression of NHEJ in mitosis by phosphorylated XRCC4. NHEJ is the major pathway for the repair of radiation and topoisomerase II poison-induced DSBs in interphase mammalian cells. CDK1 and PLK1 are activated as cells enter mitosis and contribute to phosphorylation of XRCC4 on serine 326 in the unstructured, C-terminal tail [3]. It is also likely that other protein kinases are involved in phosphorylation of XRCC4 in mitosis. In ways that are yet to be determined, phosphorylated XRCC4 suppresses C-NHEJ in mitosis, preventing formation of anaphase bridges (shown in the DAPI-stained mitotic cell in the lower panel, from [22]). Serine 326 phosphorylation of XRCC4 in mitosis is transient [3], suggesting that phosphorylated XRCC4 is either dephosphorylated or degraded as cells exit mitosis, allowing C-NHEJ to resume in the subsequent G1 phase.

What then of other repair processes in mitosis? Shinohara and colleagues report that anaphase bridge formation requires primarily the C-NHEJ pathway, as the number of bridges was decreased in cells depleted for the essential C-NHEJ protein, XRCC4. By contrast, depletion of CtIP resulted in increased anaphase bridge formation, suggesting that Alt-NHEJ or HRR suppresses anaphase bridge formation. Moreover, they show that both CDK1 and PLK1 protein kinases contribute to phosphorylation of XRCC4 in mitosis and conclude that phosphorylation of serine 326 in the extreme C-terminus of XRCC4 reduces anaphase bridge formation and inhibits DSB repair (Figure 1). The authors propose that CDK1/PLK1-dependent phosphorylation of XRCC4 serves as a switch to inhibit C-NHEJ in mitosis, preventing anaphase bridges and genomic instability [3]. These results are reminiscent of a previous study by this group in which they showed that CDK-dependent phosphorylation of the budding yeast homologue of XRCC4 (Lif1) promotes resection-mediated alternative end joining pathways, thus, in effect, suppressing NHEJ [17].

The study of Shinohara and colleagues raises interesting questions regarding the mechanism of XRCC4-mediated suppression of C-NHEJ. The authors show that DNA ligase IV, but not XRCC4, localizes to mitotic chromosomes and that failure of XRCC4 localization suppresses NHEJ. This is unexpected because in interphase, XRCC4 binds with high affinity to DNA ligase IV and, indeed, stabilizes the DNA ligase IV protein [18], [19]. Phosphorylation of XRCC4 in mitosis was not responsible for its failure to localize to mitotic chromosomes and phosphorylation did not affect the interaction of XRCC4 with DNA ligase IV or XLF. The mechanism underlying inactivation of XRCC4 function in mitosis, therefore, remains to be determined. While clearly implicating both CDK1 and PLK1 in phosphorylation of XRCC4 in mitosis, the results suggest that other protein kinases may also be involved. For example, phosphorylated XRCC4 runs as several bands on denaturing polyacrylamide gels. Though serine 326 is phosphorylated in at least two bands, inhibition of either CDK1 or PLK1 blocked serine 326 phosphorylation in only one band, suggesting that phosphorylation of XRCC4 in mitosis may be more complex than described to date.

The findings of Shinohara and colleagues also raise the question of what happens to XRCC4 at the end of mitosis. Is it dephosphorylated by protein phosphatases to allow NHEJ to proceed in the next G1 or is the phosphorylated form of XRCC4 targeted for degradation by the proteasome? Indeed, there are ample precedents for both. Protein phosphatases such as protein phosphatase 2A (PP2A) dephosphorylate multiple proteins required for entry into or exit from mitosis [20] and multiple proteins are degraded by the anaphase-promoting complex/cyclosome (APC/C) to allow mitotic exit [21]. It is also interesting to note that while XRCC4 is phosphorylated to inhibit C-NHEJ, another component of the pathway, DNA-PKcs, is required for accurate mitosis, as its down-regulation or inhibition leads to misaligned chromosomes and mitotic defects [22], [23]. DNA-PKcs is phosphorylated by PLK1 early in mitosis and is dephosphorylated by protein phosphatase 6 (PP6) at mitotic exit [22]. It will be interesting to determine how phosphorylation of DNA-PKcs and possibly other components of the C-NHEJ pathway contribute to maintenance of genome stability during mitosis. Together, these studies shed light on how DSB repair processes are regulated to prevent genomic instability during mitosis.
